# Ten simple rules for collaborative lesson development

**DOI:** 10.1371/journal.pcbi.1005963

**Published:** 2018-03-01

**Authors:** Gabriel A. Devenyi, Rémi Emonet, Rayna M. Harris, Kate L. Hertweck, Damien Irving, Ian Milligan, Greg Wilson

**Affiliations:** 1 Douglas Mental Health University Institute, McGill University, Montreal, Quebec, Canada; 2 Univ Lyon, UJM-Saint-Etienne, Saint-Étienne, France; 3 The University of Texas at Austin, Austin, Texas, United States of America; 4 The University of Texas at Tyler, Tyler, Texas, United States of America; 5 CSIRO Oceans and Atmosphere, Hobart, Tasmania, Australia; 6 University of Waterloo, Waterloo, Ontario, Canada; 7 DataCamp, Toronto, Ontario, Canada; Dassault Systemes BIOVIA, UNITED STATES

## Introduction

Lessons take significant effort to build and even more to maintain. Most academics do this work on their own, but leveraging a community approach can make educational resource development more sustainable, robust, and responsive. Treating lessons as a community resource to be updated, adapted, and improved incrementally can free up valuable time while increasing quality.

Despite the success of openness in software development and the curation of Wikipedia, it is an uncommon approach in the academic instructional setting. Each year, thousands of university lecturers teach subjects ranging from first year biology to graduate-level courses in Indian film. Some use textbooks written by one or a few authors, but beyond that they develop and maintain their course materials in isolation.

Given that academic research often depends on sharing, this differing approach to developing pedagogical materials is interesting, but the sociology and psychology behind such a blind spot are beyond the scope of this paper.

The authors have many years of experience with community-developed lessons in the context of research computing in the sciences and humanities through organizations like Software Carpentry and Programming Historian [1]. Software Carpentry was founded in 1998 to teach scientists basic computing skills and has since spawned two sibling organizations called Data Carpentry and Library Carpentry. Programming Historian was founded in 2008 and has evolved into a collaboratively edited site providing lessons to humanities scholars. Their guiding principles are that lessons should be 1) open and easily accessible as well as 2) continually maintained, refined, and improved by a community of contributors.

**Fig 1:**
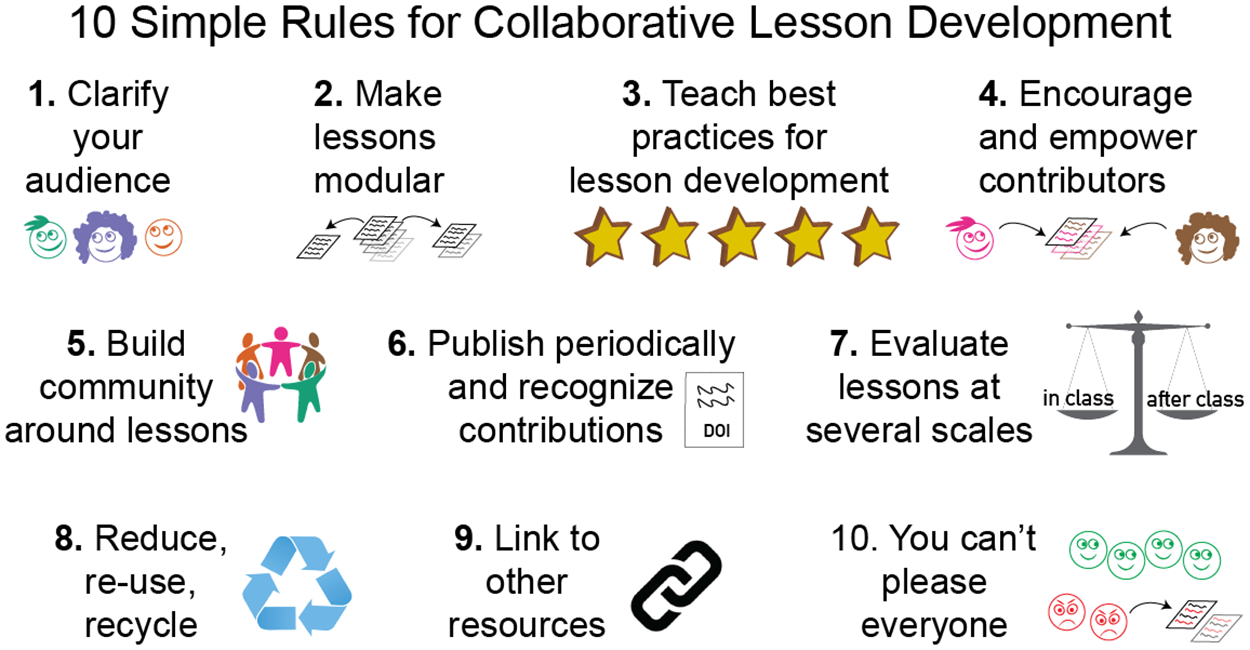
Graphical abstract of 10 simple rules for collaborative lesson development. **1.** To clarify your audience, consider writing learner profiles (Box 1). **2.** Make lessons modular by breaking them into small, single-purpose modules. **3.** Teach your instructors the best practices for developing, delivering, and maintaining lessons. **4.** Encourage and empower contributors by making the contribution process transparent and straightforward. **5** Build a community around lessons by creating opportunities for participation and mentorship. **6.** Publish new versions periodically and recognize contributors by their unique identifiers (e.g., ORCID). **7.** Evaluate lessons during and after class for a complete picture of their efficacy. **8.** Reduce, reuse, or recycle lessons before creating a new one from scratch. **9.** Link to other resources that complement the lesson content. **10.** Remember that you can’t please everyone in your audience or community.

**Fig 2:**
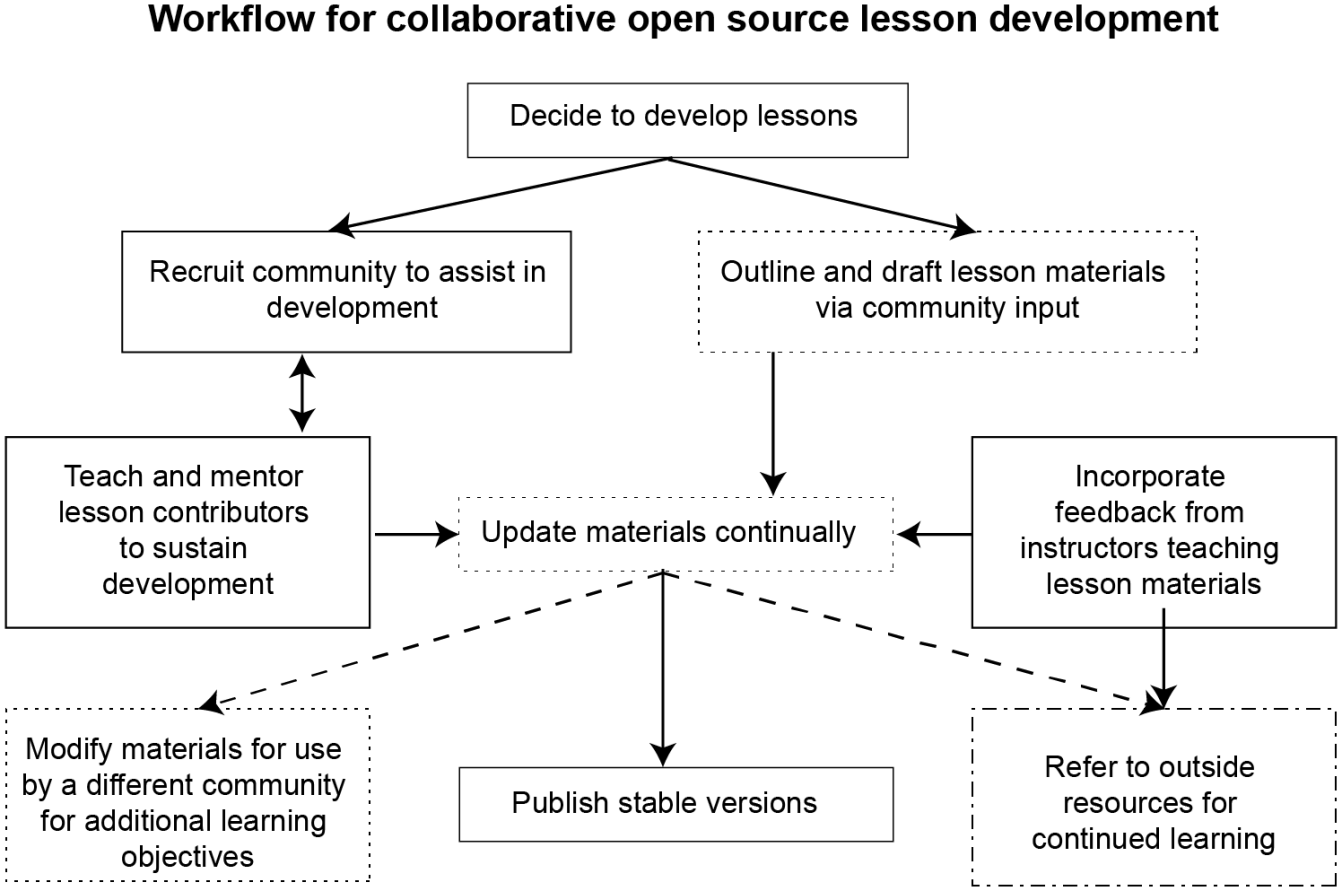
Collaborative open lesson development. Following the decision to develop lessons, activities focus on lesson development as well as community building. Boxes surrounded by dotted lines represent community contributions to lessons. Dashed arrows represent connections to activities outside the original lesson design. The box enclosed in a dashed dotted line represents unaffiliated learning resources.

All open education projects (e.g., massive open online courses) satisfy the first criterion by definition, but very few satisfy the second. In other words, while it is common for open education projects to be occasionally updated by an individual or small team (as happens when a new edition of a book is edited and published), this is not the same as continuous improvement by a large community of contributors. The 10 simple rules that follow summarize what we have learned about doing so as maintainers, editors, and reviewers of lessons used by tens of thousands of people (Fig 1). By following these rules, we contend that it is possible to create higher quality lessons than could be created by an individual or small team, both in terms of accuracy and pedagogy (Fig 2). As an added bonus, the lessons are always up-to-date and require less time per author to develop and maintain.

## Rule 1: Clarify your audience

The first requirement for building lessons together is to know for whom they are being built. "Archaeology students" is far too vague: are you and your collaborators thinking of first year students who need an introduction to the field, graduate students who intend to specialize in the subdiscipline that is the lesson’s focus, or someone in between? If different contributors believe different things about prerequisite knowledge, equipment or software required, or how much time learners will have, they will find it difficult to work together.

Instead of starting with learning objectives (Rule 3), it can be helpful to write learner profiles to clarify the learner’s general background, what they already know, what they think they want to do, how the material will help them, and any special needs they might have. This technique is borrowed from user interface design, and a typical learner profile is presented in Box 1.

**Box 1: Learner profile.**

Jorge has just moved from Costa Rica to Canada to study agricultural engineering. While fluent in both Spanish and English, he has a hearing disability that sometimes makes it hard for him to understand lectures, particularly in noisy environments. Other than using Excel, Word, and the internet, Jorge’s most significant previous experience with computers is helping his sister build a WordPress site for the family business.

Jorge needs to measure properties of soil from nearby farms using a handheld device that sends text files to his computer. Right now, Jorge has to open each file in Excel, crop the first and last points, and calculate an average. This workshop will show Jorge how to write a small Python program to read the data, select the right values from each file, and calculate the required statistics.

## Rule 2: Make lessons modular

Every instructor’s needs are different, so build small chunks that can be repurposed in many ways. A university lecturer in meteorology, for instance, might construct a course for their students by bringing together lessons on differential equations, fluid mechanics, and absorption spectroscopy. Creating courses this way shifts the instructor’s burden from writing to finding and synthesizing, which are easier if lessons clearly define what they cover (Rule 1) and if lessons have been designed by people with a shared worldview (Rule 3).

One way to achieve this is to take existing courses and break them down into smaller, single-purpose modules (a change which has pedagogical and administrative advantages in its own right). When this is done, these modules can be made more discoverable by referencing specific points in the model curricula promulgated by many professional societies (e.g., as learning objectives). Smaller modules are also more approachable for new contributors (Rule 4).

## Rule 3: Teach best practices for lesson development

Decades of pedagogical research have yielded many insights into how best to build and deliver lessons [2]. Unfortunately, many college and university faculty have little or no formal training in education [3], so this knowledge is rarely applied in the classroom.

Our experience is that even a brief introduction to a few key practices helps collaborative lesson development. If people have a shared understanding of how lessons should be developed, it is easier for them to work together. Less obviously, if people have a shared model of how lessons will be used, they are more likely to build reusable material. Finally, teaching people how to teach is a great way to introduce them to each other and build community (Fig 2).

By way of example, Software Carpentry encourages its volunteers to use the popular lesson development methodology presented by Wiggins and McTighe [4], in which learning objectives and assessments are created before any lesson materials are developed. In particular, “summative assessments” are created to determine whether the learning objectives have been met, and “formative assessments” are created to gauge the progress of learners and to give them a chance to practice key skills. The completed formative assessments are put in order and only then are the lessons written, with the aim of connecting each formative assessment to the next. This method is effective in its own right, but its greatest benefit is that it gives everyone a framework for collaboration.

An example of how to teach such pedagogical practices is Software Carpentry’s instructor training program. First offered in 2012, it is now a two-day course delivered both in person and online [5–7]. In addition to a focus on pedagogy, the course teaches whom Software Carpentry’s lessons are for, how they are delivered, and how they are maintained. Largely as a result of this training, several hundred people per year now contribute to Software Carpentry’s lessons.

## Rule 4: Encourage and empower contributors

Making the process for contributing to a lesson simple and transparent is the key to receiving contributions. Licensing, code of conduct, governance, and the review and publication process must all be explicit rather than implicit to lower the social barriers to contribution.

Tools can help, especially if they allow proposed changes to be viewed and discussed prior to their incorporation into the lessons. (In software development, this is known as “premerge review.”) However, some tools that are popular in open-source software development have considerable up-front learning costs. Portals like GitHub, for example, support everything that open lesson development needs but require contributors to use Git, which has a notoriously steep learning curve [8].

Complicating matters further, some file formats make collaboration easier or more difficult. Despite their ubiquity, open-source version control systems do not directly support review or merge of Microsoft Office or OpenDocument file formats, which raises an additional burden for newcomers [9]. While Google Docs and wikis lack some capabilities, such as full-fledged premerge review (although "suggest mode" mitigates this to some degree), their low barrier to entry makes them more welcoming to newcomers.

The best way to choose tools for managing lessons is to ask potential contributors what they are comfortable with rather than requiring them to come to you. Remember also that contributing to a lesson is probably not their top priority, and look for ways to reduce their cognitive load. For example, threaded discussion forums can improve the signal-to-noise ratio by reducing long “reply all” email exchanges. Several open frameworks are available to facilitate development of new lessons, such as learnr (https://rstudio.github.io/learnr), Morea (https://morea-framework.github.io), and DataCamp’s templates (https://www.datacamp.com/teach/documentation).

## Rule 5: Build community around lessons

Software versions and dependencies are constantly changing, while the academic literature is advancing at an ever-increasing pace. As a result, what is cutting edge one year may be out of date the next and simply wrong the year after. Collaborative lesson development groups must therefore focus on creating a community in which contributors support each other rather than relying on a small group of stewards. Authors cannot be expected to maintain continual vigilance on a lesson, but this is necessary for continual use.

A key part of doing this is to create opportunities for legitimate peripheral participation. Curating a list of small tasks that newcomers can easily tackle, encouraging them to give feedback on proposed changes, or asking them to add new exercises and tweak diagrams and references can all provide an on-ramp for people who might question their own authority or ability to change the main body of a lesson. Equally, acknowledging all contributions, however small, gives new contributors an early reward for taking part.

In 2015, Software Carpentry established a Mentoring Subcommittee to support instructors as they progress through training, teaching, and curriculum development. The Mentoring Subcommittee has promoted community building by providing virtual spaces where instructors from all over the world can share success stories and discuss strategies for overcoming challenges. This has helped strengthen the community and provided insight into how lessons can be improved (Rule 7).

Finally, working in the open can be great, but it can also unintentionally suppress voices. Programming Historian makes an ombudsperson available for private chats and facilitation to ensure that no one is excluded. Software Carpentry operates by a Code of Conduct that outlines acceptable standards of behavior for community members and those interacting with the Carpentries at events and in virtual spaces. Community members on a Policy Subcommittee serve as advocates for the Code of Conduct and adjudicate reported violations.

## Rule 6: Publish periodically and recognize contributions

Like software, specific versions of lessons should be published or released periodically so that learners or instructors have something stable to refer to for the duration of their use (Fig 2). Periodic releases also provide an opportunity for recognizing the contributions of new authors and maintainers.

Academia has only a few ways of recognizing contributions. Until these are expanded, it is important to publish lessons in ways that traditional academic systems can digest. One is to give releases DOIs supplied by providers such as Zenodo (https://zenodo.org/) or DataCite (https://www.datacite.org/). Contributors can be listed as authors and the maintainers of the lesson as editors to differentiate recognition of their contributions. Each time the lesson is published, names and identifiers such as ORCIDs (https://orcid.org) should be gathered for all contributors.

A lesson release is a good opportunity to bring the material into a stable shape by fixing outstanding issues and merging contributions. Version control automatically maintains a list of contributors and can also be used to track which content is in which release (e.g., using branches or tags). Lesson releases should use a consistent naming scheme; Software Carpentry has used the year and month of release (e.g., "2017.05") in its releases [10, 11].

If lessons are being released regularly, automate the process and archive old versions in a discoverable location. Also make sure that everyone involved knows what "done" looks like, i.e., which outstanding issues have to be addressed and how they have to be formatted in order for the next release to go out. A simple checklist stored with the lesson materials is good enough to start, but as time goes by, the community may want to use an issue tracking system of some sort so that work items can be assigned to specific people and then ticked off as they are completed.

## Rule 7: Evaluate lessons at several scales

What people immersed in developing lessons think needs fixing can easily differ from what learners think. It is therefore critical to gather and act on feedback at several scales to check assumptions and stay on course (Fig 2).

Microscale feedback can be gathered by an instructor while teaching a particular lesson. Learners can provide feedback on everything from typographical errors and the clarity of quiz questions to the order in which topics are presented, all of which the instructor should record at the end of each class in some shared location (such as a Google Doc or GitHub issues). As well as encouraging direct verbal feedback, it is a good idea to provide learners with a means to provide feedback anonymously during class (e.g., on small pieces of paper like sticky notes or through anonymous surveys).

Surveys and interviews before and after class should be used to uncover larger issues, particularly those arising from developers not fully understanding their audience, e.g., assuming prior knowledge that learners do not have. Such surveys are most effective when conducted 30–90 days after class; this gives people time to reflect, so their feedback will more accurately reflect what they learned rather than how entertained they were. Clearly stated learning objectives (Rule 3) are essential here, as they tell assessors what they should be measuring.

## Rule 8: Reduce, reuse, recycle

Just as a scholar would not write a paper without a literature review, an instructor should not create a new lesson if there is an existing one they could use or contribute to. A short online search can reveal if someone has written what you need, whether it is complementary to your goals, and if it can be tweaked or modified to meet your needs.

Before reusing content, make sure to check its license. Both Programming Historian and the Carpentry projects use the Creative Commons–Attribution license (https://creativecommons.org/licenses/by/4.0/), which allows people to share and adapt material for any purpose as long as they cite the original source. Other Creative Commons licenses may restrict commercial use and/or creation of derivative materials.

The question of licensing also arises when recycling lesson components such as images, data, figures, or code. If the license does not cover them explicitly, ask permission as you would for any other academic material.

The converse of this rule is to make the license for your lessons explicit and discoverable. For example, when lessons are published (Rule 6), make sure that keywords such as "CC-BY" appear in their bibliography entries and HTML page headers.

## Rule 9: Link to other resources

Learners are unlikely to absorb everything they need to know about a topic from your lesson alone. This is partly a matter of scope—any interesting subject is too large to fit in a single lesson—but also a matter of level and direction. As Caulfield has argued [12], the best way to use the internet is to provide a chorus of explanations that offer many angles and approaches for a given topic, each of which may be the best fit for a different set of needs (Fig 2).

Collaboratively developed lessons should direct learners to these resources at strategic points. If a community or discussion forum exists for the topic, such as textbooks, technical documentation, videos, web pages, threads on Quora, or mailing lists, then it is worth including.

Doing this is substantial work, and maintaining it even more so, which makes building community around lessons (Rule 5) all the more important. In particular, it is vital to engage the learners as equal participants in that community. They should be able to propose updates, corrections, and additions to lessons and know that they are encouraged to do so (Rule 4).

## Rule 10: You can’t please everyone

No single lesson can be right for every learner. Two people with no prior knowledge of a specific subject may still be able to move at different speeds because of different levels of general background knowledge. Similarly, lessons on ecology for learners in Utah and Vietnam will probably be most relatable if they use different examples. A community may therefore maintain several differently oriented or differently paced lessons on a single topic, just as programming languages provide several different libraries for doing the same general thing with different levels of performance and complexity.

Similarly, no lesson development community can serve all purposes. Some groups may prioritize rapid evolution, while others may prefer a "measure twice, cut once" approach. If there are complementary ways to explain something or points of view that can cohabit respectfully, it may be possible to present them side by side. There are good pedagogical reasons to do this even if contributors do not disagree: weighing alternatives fosters higher-order thinking.

But sometimes choices must be made. The open-source software community has wrestled with these issues for three decades and has evolved some best practices to address them [13]. As discussed in Rule 4, the first step is to have a clear governance structure and a clear, permissive license. Minor disagreements should be discussed openly and respectfully. If they turn out not to be so minor after all, contributors should split off and evolve the lesson in the way they see best. (This is one of the reasons to have a permissive license.)

These splits rarely happen in practice. When they do, it is important to remember that we all share the same vision of better lessons built together.

## Conclusion

Every day, teachers all over the world spend countless hours duplicating each other’s work. These 10 rules provide an alternative: adopting the model of collaborative software development to make more robust and sustainable lessons in all domains that can be continually improved by those who use them. We hope that our experiences can help others teach more with more impact and less effort.
